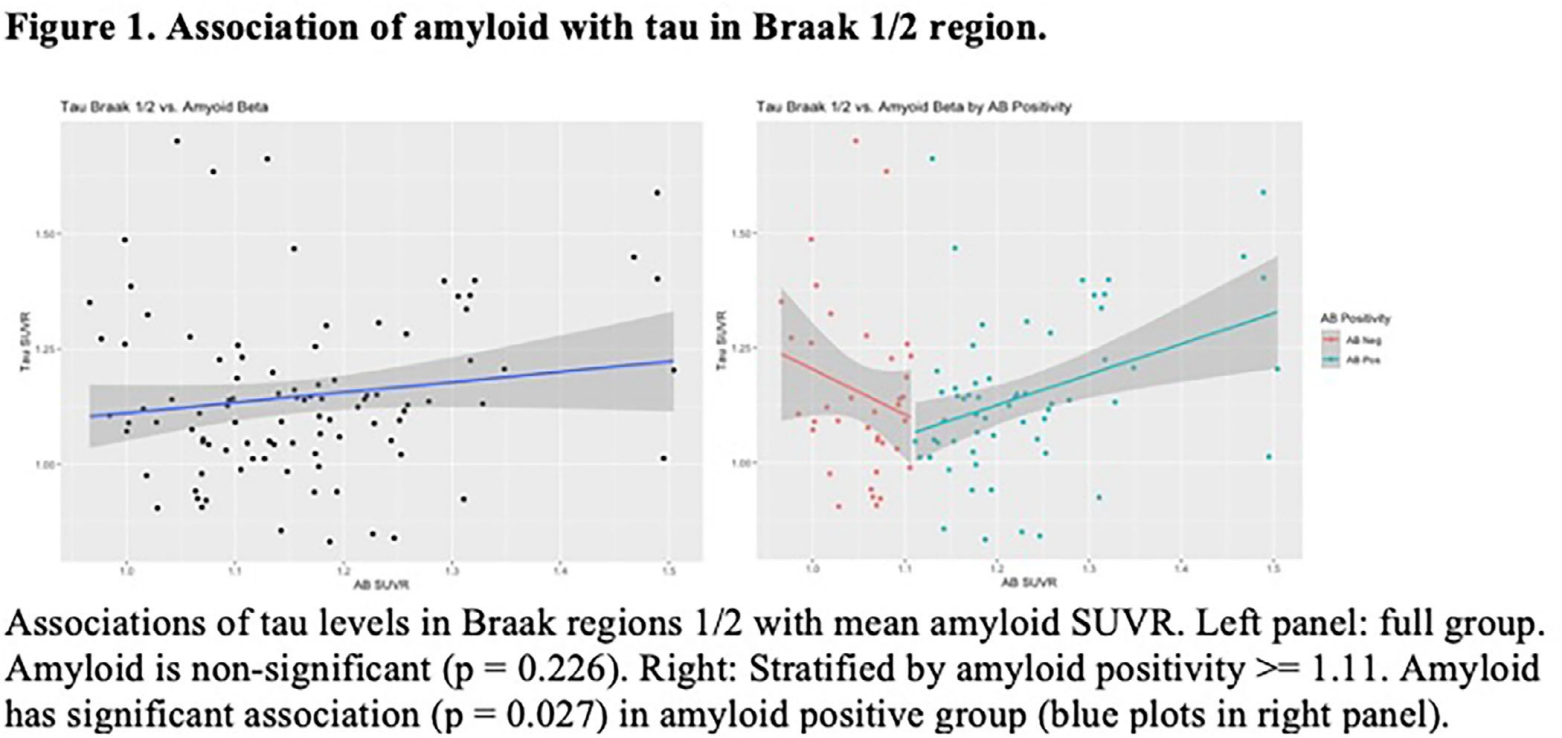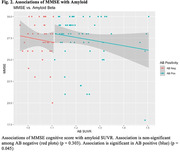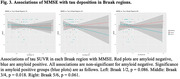# Associations of cerebral amyloid and tau with neurocognitive performance: Findings from the 90+ Study

**DOI:** 10.1002/alz.093059

**Published:** 2025-01-09

**Authors:** Evan Fletcher, Shraddha Sapkota, Oliver Martinez, María M. M. Corrada, Davis C. Woodworth, S. Ahmad Sajjadi, Charles Decarli

**Affiliations:** ^1^ UC Davis, Davis, CA USA; ^2^ University of California, Davis, Davis, CA USA; ^3^ University of California, Irvine, Irvine, CA USA

## Abstract

**Background:**

Associations between cognition and brain markers of amyloid, tau, and neurodegeneration are poorly understood in the oldest‐old (>90 years) compared to younger populations. Prior work suggests that the link between neuropathology and cognition may become weaker in advanced old age, reducing the applicability of accepted biomarker cascade models of Alzheimer’s disease progression. Addressing this question in the oldest‐old is challenging, partly due to limited data and methodological challenges of neuroimaging analysis. In this study, we examine the associations of (1) cerebral amyloid and tau, (2) amyloid and cognitive performance, and (3) tau and cognitive performance, in the overall group and as stratified by amyloid positivity.

**Method:**

Participants were from the oldest‐old cohort from the 90+ Study at University of California, Irvine (N=98; mean age at assessment=92.04 years; %female=53,). We used MRI‐free analyses of PET imaging to investigate the relations among amyloid (AV45), tau (AV1451) in Braak regions 1/2, 3/4 and 5/6, and cognitive measure (Mini‐Mental State Exam [MMSE]). Native PET images were warped directly to an appropriate PET template, yielding SUVRs computed using cerebellar reference means. Amyloid positivity used an SUVR cutoff of 1.11, computed over a standard index region. All regressions were controlled for age, sex, education, and Apolipoprotein E status.

**Result:**

Group characteristics are in Table 1. In the amyloid positive group (N=61), we found significant associations for amyloid with tau in Braak 1/2 (p=0.03; Figure 1), and MMSE (p=0.045; Figure 2). Associations of MMSE with tau Braak regions varied (Braak 1/2: p=0.086, Braak 3/4: p=0.018, Braak 5/6: p=0.061; Figure 3). We observed no significant associations in the full cohort or in the amyloid negative group.

**Conclusion:**

MRI‐free PET processing yielded results in the oldest‐old that are consistent with expected relations among amyloid, tau and cognition. Accurate SUVR measurement requires rigorous template registration and quality control. All significant associations occurred in the amyloid positive group, showing expected relations of amyloid to cognition and early Braak stage tau, and tau to cognition, consistent with accepted models. However, small sample size limited our findings. Further studies are needed to clarify the applicability of amyloid‐tau models in this age group.